# qSOFA score poorly predicts critical progression in COVID-19 patients

**DOI:** 10.1007/s10354-021-00856-4

**Published:** 2021-06-29

**Authors:** Sven Heldt, Matthias Neuböck, Nora Kainzbauer, Guangyu Shao, Thomas Tschoellitsch, Martin Duenser, Bernhard Kaiser, Markus Winkler, Christian Paar, Jens Meier, Bernd Lamprecht, Helmut J. F. Salzer

**Affiliations:** 1 Department of Pulmonary Medicine, Kepler University Hospital, Krankenhausstraße 9, 4021 Linz, Austria; 2 Department of Anesthesiology and Intensive Care Medicine, Kepler University Hospital, Linz, Austria; 3 Department of Pathology and Microbiology, Kepler University Hospital, Linz, Austria; 4 Institute of Laboratory Medicine, Kepler University Hospital, Linz, Austria

**Keywords:** SARS-CoV-2, Organ dysfunction scores, Austria, Death, Intensive care unit, SARS-CoV-2, Score für Organdysfunktion, Österreich, Tod, Intensivstation

## Abstract

**Background:**

In December 2019, the new virus infection coronavirus disease 2019 (COVID-19) caused by the severe acute respiratory syndrome coronavirus 2 (SARS-CoV-2) emerged. Simple clinical risk scores may improve the management of COVID-19 patients. Therefore, the aim of this pilot study was to evaluate the quick Sequential Organ Failure Assessment (qSOFA) score, which is well established for other diseases, as an early risk assessment tool predicting a severe course of COVID-19.

**Methods:**

We retrospectively analyzed data from adult COVID-19 patients hospitalized between March and July 2020. A critical disease progress was defined as admission to intensive care unit (ICU) or death.

**Results:**

Of 64 COVID-19 patients, 33% (21/64) had a critical disease progression from which 13 patients had to be transferred to ICU. The COVID-19-associated mortality rate was 20%, increasing to 39% after ICU admission. All patients without a critical progress had a qSOFA score ≤ 1 at admission. Patients with a critical progress had in only 14% (3/21) and in 20% (3/15) of cases a qSOFA score ≥ 2 at admission (*p* = 0.023) or when measured directly before critical progression, respectively, while 95% (20/21) of patients with critical progress had an impairment oxygen saturation (SO_2_) at admission time requiring oxygen supplementation.

**Conclusion:**

A low qSOFA score cannot be used to assume short-term stable or noncritical disease status in COVID-19.

## Abstract (de)

**Grundlagen:**

Im Dezember 2019 kam es zum Auftreten der neuen Virusinfektion „coronavirus disease 2019“ (COVID-19), hervorgerufen durch das „severe acute respiratory syndrome coronavirus 2“ (SARS-CoV-2). Einfache klinische Scores zur frühzeitigen Risikostratifizierung könnten das Management von COVD-19-Patient(inn)en verbessern. Ziel dieser Pilotstudie war es daher, den für andere Erkrankungen etablierten qSOFA-Score („quick sequential organ failure assessment score“) als frühzeitige Risikobewertung für kritische Krankheitsverläufe bei COVID-19 zu evaluieren.

**Methodik:**

Es erfolgte eine retrospektive Datenanalyse von hospitalisierten COVID-19-Patient(inn)en aus dem Zeitraum März bis Juli 2020. Ein kritischer Krankheitsverlauf wurde als Aufnahme auf die Intensivstation (ICU) oder Tod definiert.

**Ergebnisse:**

Von 64 COVID-19-Erkrankten wiesen 33 % (21/64) einen kritischen Krankheitsverlauf auf, wovon 13 Patient(inn)en auf die ICU verlegt wurden. Die COVID-19-assoziierte Sterblichkeitsrate betrug 20 % und stieg nach ICU-Aufnahme auf 39 % an. Bei allen Patient(inn)en ohne kritischen Verlauf war bei Aufnahme der qSOFA-Score ≤ 1. Patient(inn)en mit einem kritischen Verlauf hatten in nur 14 % (3/21) der Fälle bei Aufnahme (*p* = 0,023) bzw. in 20 % (3/15) der Fälle direkt vor kritischer Verschlechterung einen qSOFA-Score ≥ 2, wohingegen 95 % (20/21) der Patienten und Patientinnen mit einem kritischen Verlauf zum Zeitpunkt der Aufnahme eine reduzierte Sauerstoffsättigung (SO_2_) aufwiesen, mit der Notwendigkeit der zusätzlichen Sauerstoffgabe.

**Schlussfolgerung:**

Bei einem niedrigen qSOFA-Score kann nicht von einem kurzfristig stabilen oder unkritischen Krankheitsverlauf von COVID-19 ausgegangen werden.

## Introduction

At the end of December 2019, several cases of an ominous acute respiratory disease were reported from Wuhan city in China. A new strain of a contagious coronavirus was identified called severe acute respiratory syndrome coronavirus 2 (SARS-CoV-2) causing coronavirus disease 2019 (COVID-19). Within a few weeks the virus spread to other countries putting the global community on alert. On March 11, 2020, the World Health Organization (WHO) officially declared the emerging spread of SARS-CoV‑2 as a pandemic.

Risk scores predicting severe courses of COVID-19 could be useful leading to early and more intense monitoring. This would be important to initiate necessary intensive medical measures at an early stage, to look closer on other effected organ systems than the respiratory system (especially for affected renal function, neurologic impairments, and myocardial involvement [1]) and thus to initiate the necessary instrumental diagnostics, which would otherwise be used rather cautiously due to the risk of infection.

With the redefinition for sepsis and septic shock in 2016 a new bedside clinical score termed quick Sequential Organ Failure Assessment (qSOFA) was recommended to identify infected patients at high risk for bad outcomes. Other than the more extensive original SOFA score it provides simple criteria using only alteration in mentation, hypotension (systolic blood pressure of 100 mm Hg or less) and increased respiratory rate (≥ 22/min). A score ≥ 2 was recommended as an indicator for potential sepsis in patients with suspected infection outside the intensive care unit (ICU) and was found to be as accurate as the SOFA score in predicting their mortality [2]. Jiang et al. [3] found a qSOFA score ≥ 2 being strongly associated with mortality in patients with pneumonia including six studies with 17,868 patients in a meta-analysis in 2018.

However, due to the threatened capacities of health care systems worldwide during the COVID-19 pandemic, there is a strong need for a validated clinical risk score to identify patients with confirmed SARS-CoV‑2 infection at risk for severe or critical disease progression. Due to its ease of access requiring no laboratory tests, qSOFA could be a valuable tool for risk stratification in patients with COVID-19 and help clinicians with the decision upon start or escalation of therapy and the consideration of referring patients to the intensive care unit. Its predictive value for mortality and critical progression in patients with COVID-19, however, has not been sufficiently evaluated so far.

The aim of this pilot study was to ascertain the value of the qSOFA score as a first screening tool for a risk of critical disease progression in hospitalized COVID-19 on the day of admission. Critical disease progression was defined as ICU admission or death.

## Materials and methods

### Study setting and design

We retrospectively analyzed data from polymerase chain reaction (PCR)-confirmed COVID-19 patients hospitalized at Kepler University Hospital in Linz (KUK), Austria, between March 5 and July 23, 2020.

The study was approved by the local ethics committee of Upper Austria (approval number [EK Nr] 1085/2020). We included adult patients who were admitted to the hospital not requiring ICU initially (= normal ward) and had a positive SARS-CoV‑2 real-time polymerase chain reaction (PCR) test. Every test result from a respiratory sample was considered, including naso-/pharyngeal swab, tracheal secretion or bronchoalveolar lavage.

### Definitions and qSOFA score

Critical disease progression was defined as being transferred from the normal ward to the ICU or death. The qSOFA score considers (a) a Glasgow coma scale value smaller than 15, (b) a respiratory rate ≥ 22/min and (c) a systolic blood pressure of ≤ 100 mm Hg. The highest score (max. 3 points) is associated with poor prognosis. These qSOFA scores were collected 3–5 times daily during clinical routine, depending on the patient’s general condition. We used the worst score of day 0 and the last documented score before the critical event (ICU transfer or death) for the analysis.

### Molecular SARS-CoV-2 testing

SARS-CoV‑2 PCR analyses were routinely performed with the fully automated Cobas 6800 system (Roche, Mannheim, Germany). Selective amplification of target nucleic acid from the sample is achieved using target-specific forward and reverse primers for ORF1a/b nonstructural region that is unique to SARS-CoV‑2. Additionally, a conserved region in the structural protein envelope E‑gene is used for pan-Sarbecovirus detection, which also detects SARS-CoV‑2. A tested sample is considered positive if any of the aforementioned target genes returns a positive result as indicated by a target specific Ct-value.

Alternatively, especially before the introduction of the SARS-CoV‑2 test on the Cobas 6800 instrument, viral RNA was extracted either with MagNA Pure total nucleic acid large volume kit (Roche, Mannheim, Germany) or with the QIAsymphony virus/pathogen midi kit (Qiagen, Hilden, Germany). PCR analyses were subsequently performed with the COVID-19 PCR kit (Genesig, Camberley, UK) utilizing the RdRP gene as a single target assay on the Cobas z 480 instrument (Roche, Mannheim, Germany).

### Data collection and statistical methods

Patient data collected included demographics including body mass index (BMI), comorbidities, smoking status, symptoms, symptom onset, time interval between symptom onset and ICU transfer and/or death, time interval between hospital admission and ICU transfer and/or death, radiological course, and vital signs including blood pressure, Glasgow Coma Scale (GCS), respiratory rate, and pulsoxymetric peripheral oxygen saturation. These data were routinely collected during clinical care and documented in the electronic patient file. Retrospectively, they were transferred to a MS Excel spreadsheet version 2010 (Microsoft, Redmond, WA, USA) by one of the authors and crosschecked by another one.

We performed data analysis with R‑4.0.2. We used Χ^2^ test and fisher’s exact test for cross tables to check for differences in categorical variables. To identify differences in metric variables we used the nonparametric Mann–Whitney U test respectively the t‑test. The significance level was set at *p* ≤ 0.05.

## Results

### Baseline characteristics and outcomes

Between March 5th and July 23rd, 64 patients (30 female, 34 male) with laboratory confirmed COVID-19 were hospitalized at the department for pulmonary medicine at the Kepler University Hospital in Linz. The median age was 70 years (27–93 years). Baseline characteristics are shown in Table 1. There were no statistically significant differences in sex, age distribution, BMI, or typical radiologic findings for COVID-19 in X‑rays. There was no difference in the worsening of radiological findings.

**Table 1:** Baseline characteristics comparing patients without and with critical disease progression. Critical disease progression is defined as either deceased or intensive care unit (ICU) admission

	All (*n* = 64)	Without critical progression (*n* = 43)	With critical progression (*n* = 21)	*p*-value
*Sex*				0.934
Male [*n* (%)]	34 (53.1)	23 (53.5)	11 (52.4)	–
Female [*n* (%)]	30 (46.9)	20 (46.5)	10 (47.6)	
*Age* *[median (min–max)]*	70.0 (27.0–93.0)	70.0 (38.0–93.0)	74.0 (27.0–93.0)	0.431
*Age in categories* *[n (%)]*				0.857
< 50	10 (15.6)	7 (16.3)	3 (14.3)	–
50–59	9 (14.1)	6 (14.0)	3 (14.3)	
60–69	10 (15.6)	8 (18.6)	2 (9.5)	
70–79	18 (28.1)	12 (27.9)	6 (28.6)	
≥ 80	17 (26.6)	10 (23.3)	7 (33.3)	
*BMI [n (%)]* *(2 missing)*				0.912
< 18.5	4 (6.5)	3 (7.1)	1 (5.0)	–
18.5–24.9	16 (25.8)	10 (23.8)	6 (30.0)	
24.9–29.9	31 (50.0)	22 (52.4)	9 (45.0)	
≥ 30	11 (17.7)	7 (16.7)	4 (20.0)	
*Smoking [n (%)]*				
Current	1 (1.6)	1 (2.3)	0 (0)	0.481
Former	14 (21.9)	8 (18.6)	6 (28.6)	0.365
*Comorbidities [n (%)]*				
Chronic lung disorder	6 (9.4)	4 (9.3)	2 (9.5)	0.977
Arterial hypertension	32 (50.0)	21 (48.8)	11 (52.4)	0.791
Diabetes	15 (23.4)	6 (14.0)	9 (42.9)	**0.010**
Active cancer	7 (10.9)	2 (4.7)	5 (23.8)	**0.021**
Atrial fibrillation	9 (14.1)	6 (14.0)	3 (14.3)	0.971
Chronic kidney disease	11 (17.2)	6 (14.0)	5 (23.8)	0.326
Coronary heart disease	9 (14.1)	5 (11.6)	4 (19.1)	0.423
*Symptoms [n (%)]*				
Fever	45 (70.3)	29 (67.4)	16 (76.2)	0.472
Dry cough	36 (56.3)	28 (65.1)	8 (38.1)	**0.041**
Chills	11 (17.2)	11 (25.6)	0 (0)	**0.011**
Fatigue	23 (35.9)	17 (39.5)	6 (28.6)	0.391
Diarrhea	14 (21.8)	8 (18.6)	6 (28.6)	0.365
*X‑Ray [n (%)]*				
Initial, unremarkable	17 (26.6)	14 (32.6)	3 (14.3)	0.120
Initial, typical infiltrates	30 (46.9)	17 (39.5)	13 (61.9)	0.092
Follow-up deteriorated	24 (70.6)	11 (57.9)	13 (86.7)	0.068

Significant *p*-values (*p* ≤ 0.5) are bold. *BMI* body mass index

One out of 64 patients was an active tobacco smoker, while 63 patients were nonsmokers. Of these 63 nonsmoking patients, 14 had a history of smoking, which was not significantly more frequent in the critical progression group (8 vs. 6; *p* = 0.365). In cases with critical progression, diabetes and active cancer were significantly more frequent (Table 1). The most common symptoms at admission were fever, dry cough, tiredness, weariness, fatigue, diarrhea, and chill. Statistically significant differences were found for occurrence of chills and dry cough; they were less frequent in patients who had to be treated in the ICU or who died. The duration of fever was not significantly different.

Patients with a critical disease progression had a significantly higher interleukin‑6 (IL‑6; *p* = 0.007), C‑reactive protein (CRP; *p* = 0.006), procalcitonin (PCT; *p* = 0.002), lactate dehydrogenase (LDH; *p* = 0.018), liver enzymes (*p* = 0.003), N‑terminal prohormone of brain natriuretic peptide (NT-proBNP; *p* = 0.022), creatinine (*p* = 0.017) and potassium level (*p* = 0.031) on the day of admission.

Patients with a critical course of COVID-19 disease were treated longer at hospital than patients without critical progression (Table 2). Of 64 hospitalized COVID-19 patients 13 (20%) had to be transferred to ICU. Reasons for transfer were respiratory deterioration in 11 patients and hemodynamic instability in 2 patients. Median time point of ICU transfer was 4 days after hospital admission and 10 days after symptom onset. Overall mortality rate was 20% (13/64 patients). Median time of death was 14 days after hospital admission and 17 days after symptom onset, respectively. COVID-19-associated ICU mortality rate was 39% (5/13).

**Table 2:** Outcomes comparing patients without and with critical disease progression. Critical disease progression is defined as either death or ICU admission

	All (*n* = 64)	Without critical progression (*n* = 43)	With critical progression (*n* = 21)	*p*-value
*qSOFA at hospital admission [n (%)]*				0.023
0	55 (85.9)	40 (93.0)	15 (71.4)	–
1	6 (9.4)	3 (7.0)	3 (14.3)	
2	0 (0.0)	0 (0.0)	0 (0.0)	
3	3 (4.7)	0 (0.0)	3 (14.3)	
*qSOFA before critical progression at normal ward (n* *=* *15) [n (%)]*				n.a.
0	n.a.	n.a.	9 (60.0)	–
1	n.a.	n.a.	3 (20.0)	
2	n.a.	n.a.	2 (13.3)	
3	n.a.	n.a.	1 (6.7)	
*Days at hospital [median (min–max)]*	11 (1–57)	9 (3–29)	17 (1–57)	0.005
*Admission to ICU [n (%)]*				n.a.
Yes	13 (20.3)	n.a.	13 (61.9)	–
No	51 (79.7)	n.a.	8 (38.1)	
*Days from hospital to ICU admission [median (min–max)]*	n.a.	n.a.	4 (1–7)	–
*Days at ICU [median (min–max)]*	n.a.	n.a.	11 (3–26)	n.a.
*Death [n (%)]*				n.a.
Yes	13 (20.3)	n.a.	13 (61.9)	–
*Yes, at normal ward*	8 (12.5)	n.a.	8 (38.1)	
*Yes, at ICU*	5 (7.8)	n.a.	5 (23.8)	
No	51 (79.7)	n.a.	8 (38.1)	
*Days from symptom onset to death [median (min–max)]*	n.a.	n.a.	17 (6–31)	n.a.
*Days from hospital admission to death [median (min–max)]*	n.a.	n.a.	14 (1–51)	n.a.
*SO*_*2*_* and oxygen supplementation* *[n (%)]*				< 0.001
SO_2_ > 94%	20 (31.3)	19 (44.2)	1 (4.8)	–
SO_2_ 90–94%	6 (9.4)	6 (14.0)	0 (0)	
Oxygen supplementation received	38 (59.4)	18 (41.9)	20 (95.2)	

*qSOFA* quick Sequential Organ Failure Assessment, *ICU* intensive care unit, *n.a.* not applicable, *SO*_*2*_ peripheral oxygen saturation

Autopsies indicated secondary acute bronchopneumonia and/or thrombotic/thromboembolic complications including microthrombi in small arterioles and thrombi in mid-sized pulmonary arteries as major causes of death [4].

### qSOFA score and diagnostic performance

**Fig. 1:**
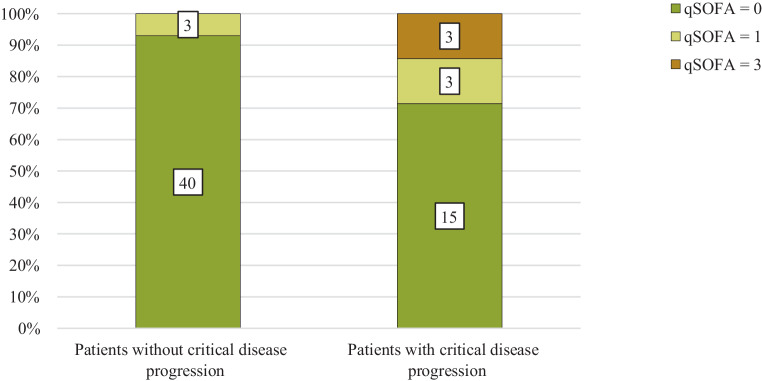
Percentage of quick Sequential Organ Failure Assessment (qSOFA) scores at the time of hospital admission in patients without and with critical progression of coronavirus disease 2019 (COVID-19) defined as intensive care unit (ICU) admission and/or death. Numbers within the bars show the number of cases

qSOFA score results differed significantly between groups “without critical progress” and “with critical progress” (*p* = 0.023; Table 2). Only patients with a qSOFA score ≥ 2 at admission experienced critical disease progress during the study (Table 2, Fig. 1). This resulted in a 100% specificity and a positive predictive value (PPV) of 100% for predicting critical disease progression (Table 3). The sensitivity was 14% as 3/21 patients with a critical progression had a score of ≥ 2 at admission. The negative likelihood ratio (LR−) was 0.86.

**Table 3:** Performance of the qSOFA score at hospital admission to predict critical disease progression in coronavirus disease 2019 (COVID-19)

All (*n* = 64)	qSOFA 2–3 (*n* = 3)	qSOFA 0–1 (*n* = 61)	
*Critical disease progression (n* *=* *21)*	3	10	Sensitivity = 14.3% (3/21)
*Without critical disease progression (n* *=* *43)*	0	43	Specificity = 100% (43/43)
**–**	PPV = 100% (3/3)	NPV = 70.5% (43/61)	–
**–**	LR+ = n.a.	LR− = 0.86	–

*qSOFA* quick Sequential Organ Failure Assessment, *n.a.* not applicable, *PPV* positive predictive value, *NPV* negative predictive value, *LR+* positive likelihood ratio, *LR− *negative likelihood ratio

When considering only the outcome “death during hospitalization”, the qSOFA score from the day of admission yields similar performance results (Table 4): Specificity and PPV were 100% and LR− was 0.77.

**Table 4:** Performance of the qSOFA score at hospital admission to predict death in coronavirus disease 2019 (COVID-19)

All (*n* = 64)	qSOFA 2–3 (*n* = 3)	qSOFA 0–1 (*n* = 61)	
*Died (n* *=* *13)*	3	10	Sensitivity = 23.1% (3/13)
*Survived (n* *=* *51)*	0	51	Specificity = 100% (51/51)
**–**	PPV = 100% (3/3)	NPV = 83.6% (51/61)	–
**–**	LR+ = n.a.	LR− = 0.77	–

*qSOFA* quick Sequential Organ Failure Assessment, *PPV* positive predictive value, *NPV* negative predictive value, *LR+* positive likelihood ratio, *LR−* negative likelihood ratio, *n.a.* not applicable

**Fig. 2:**
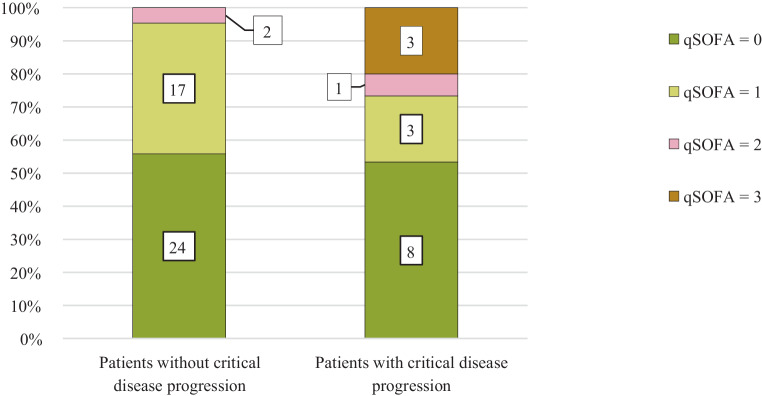
Highest qSOFA scores within the first 7 days of hospitalization on the normal ward in coronavirus disease 2019 (COVID-19) cases with and without critical progression. Only patients with a deterioration within 7 days after admission were included. The highest qSOFA scores of the first 7 days on the normal ward were chosen. Critical progression of COVID-19 was defined as intensive care unit (ICU) admission and/or death. Numbers within the bars show the number of cases. *qSOFA* quick Sequential Organ Failure Assessment

For evaluating the diagnostic performance of qSOFA scores not only at the admission day, but also within the first week of treatment and directly before critical progression, 6 cases with a critical outcome were not included. This was because of a fatal outcome after active COVID-19 disease > 14 days after hospital admission (*n* = 4) and missing information (*n* = 2). When considering the highest qSOFA scores at the normal ward within the first week, the sensitivity improved while the specificity deteriorated only slightly (Tables 5 and 6). The PPV dropped noticeably. qSOFA scores of 3 again occurred only in the group that critically deteriorated over time, but scores of 2 were distributed across both groups (Fig. 2). A qSOFA score of ≥ 2 within the first week of treatment showed a positive likelihood ratio (LR+) of 7.27 for the outcome “death” and an LR+ = 5.68 for “ICU admission and/or death”, respectively (Tables 5 and 6); a qSOFA score of ≤ 1 had an LR− of 0.61 and 0.77, respectively.

**Table 5:** Performance of normal ward qSOFA scores within the first 7 days of hospitalization to predict critical disease progression in coronavirus disease 2019 (COVID-19). Only patients with a deterioration within 7 days after admission were included. The highest qSOFA score of the first 7 days at normal ward was chosen

All (*n* = 58)	qSOFA 2–3 (*n* = 6)	qSOFA 0–1 (*n* = 52)	
*Critical disease progression (n* *=* *15)*	4	11	Sensitivity = 26.7% (4/15)
*Without critical disease progression (n* *=* *43)*	2	41	Specificity = 95.3% (41/43)
**–**	PPV = 66.7% (4/6)	NPV = 78.8% (41/52)	–
**–**	LR+ = 5.68	LR− = 0.77	–

*qSOFA* quick Sequential Organ Failure Assessment, *PPV* positive predictive value, *NPV* negative predictive value, *LR+* positive likelihood ratio, *LR−* negative likelihood ratio

**Table 6:** Performance of normal ward qSOFA scores within the first 7 days of hospitalization to predict death in coronavirus disease 2019 (COVID-19). Only patients who died within 7 days after admission were included. The highest qSOFA score of the first 7 days on the normal ward was chosen

All (*n* = 58)	qSOFA 2–3 (*n* = 6)	qSOFA 0–1 (*n* = 52)	
*Died (n* *=* *7)*	3	4	Sensitivity = 42.9% (3/7)
*Survived (n* *=* *51)*	3	48	Specificity = 94.1% (48/51)
**–**	PPV = 50.0% (3/6)	NPV = 92.3% (48/52)	–
**–**	LR+ = 7.27	LR− = 0.61	–

*qSOFA* quick Sequential Organ Failure Assessment, *PPV* positive predictive value, *NPV* negative predictive value, *LR+* positive likelihood ratio, *LR−* negative likelihood ratio

**Fig. 3:**
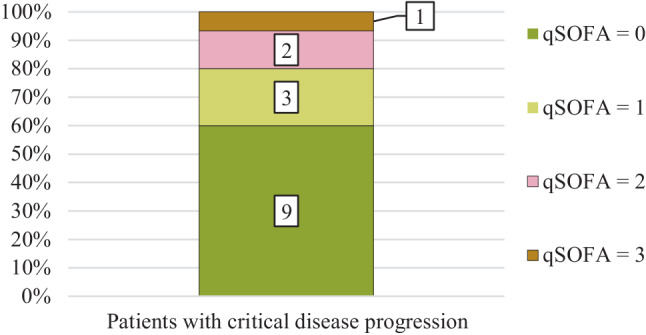
qSOFA scores measured directly before critical progression of coronavirus disease 2019 (COVID-19) defined as intensive care unit (ICU) admission and/or death. Numbers within the bars show the number of cases. *qSOFA* quick Sequential Organ Failure Assessment

In all, 23% (3/15) of cases with a critical progression within the first 7 days at hospital showed a qSOFA score ≥ 2 directly before that event (Fig. 3).

### Impaired oxygen saturation

**Fig. 4:**
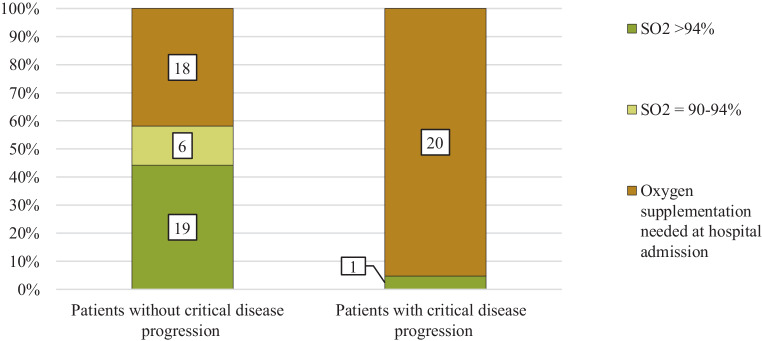
Oxygen saturation or need of oxygen supplementation at the time of hospital admission in patients without and with critical progression of coronavirus disease 2019 (COVID-19) defined as Intensive care unit (ICU) admission and/or death. Numbers within the bars show the number of cases. *SO*_*2*_ peripheral oxygen saturation

Looking at the peripheral oxygen saturation (SO_2_) shows that 95% (20/21) of the later deteriorating patients had SO_2_ impairment which did indicate oxygen supplementation at hospital admission (Table 2, Fig. 4). Although patients without ICU admission or death also had impaired peripheral oxygen saturations, only 42% (18/43) required oxygen on hospital admission. This difference was statistically significant (*p* < 0.001).

At our hospital oxygen supplementation practice usually leads to a supplementation when SO_2_ decreases to 93% or lower, and ultimately always when less than 90%.

## Discussion

Our study results indicate that the qSOFA score poorly predicts critical disease progression in COVID-19 patients. Regarding the high specificity and PPV, patients with a high qSOFA score on hospital admission do have a remarkably high risk of death and of course should therefore receive intensive care. This finding is unsurprising and not the reason for us calling the qSOFA score a poor prediction tool for critical progression in COVID-19.

However, we also calculated the performance for the worst qSOFA scores on the normal ward within the first week and included only patients with a deterioration within 7 days after admission, supporting an accurate interpretation of the qSOFA score regarding critical progress and acute viral infection. In this setting, the performance for predicting a necessary ICU transfer and/or death during the inpatient stay was not as good as the score performance at admission, but it was still acceptable.

Beside the possibility of a high score predicting a critical progression, it is more interesting that of the 21 persons with a later critical progress only 14% had a score of 2 or higher at admission. Of the patients who deteriorated during hospitalization 71% had a qSOFA score of 0 on admission, and necessary ICU admission happened 1–7 days after that point anyways. With an LR− = 0.86, a qSOFA score of ≤ 1 at admission is not a good predictor of a noncritical course. A low qSOFA score does not allow us to assume that the situation will be stable or at least not lead to necessary ICU treatment or death in the following days. It should be emphasized that even immediately before critical deterioration within the first week occurred, only 20% of the corresponding patients had a qSOFA score of ≥ 2.

Because of the casual association of a high qSOFA score with the outcome “ICU admission”, we also looked at the qSOFA score performance for only predicting death. The results were similar in that the score was also not a good predictor of surviving COVID-19.

The critical situations seemed to rather be predicted by impaired peripheral oxygen saturation. While not always associated with critical progression, it appears to be a much better indicator of which patients should be more closely assessed at hospital admission, than the qSOFA score. This is also in line with the current recommendations for hospital admission in COVID-19 patients, which focus on the respiratory impairment and considering underlying risk factors at the same time [1].

However, the calculated diagnostic performance values must be put into perspective as the number of cases in this pilot study was low and one should look critically at the calculated values. This is also true for the *p*-values with the significant different qSOFA score distribution between the groups. It is relativized by the explanations above regarding the practical application in clinical routine.

Contrary to the expected uneven distributions between the progressive and nonprogressive groups with respect to the known risk factors for a more severe course, these inequalities were mostly not evident in our cohort. Statistical differences in the comparison of the baseline characteristics of the two groups resulted on the one hand in the pre-existing conditions of diabetes mellitus type 2 and cancer. This observation is not surprising and in line with already published data, since these are already known risk factors for a more severe course of COVID-19. However, pre-existing arterial hypertension, which is also considered to be a particularly relevant risk factor, as well as chronic lung, kidney, or heart disease, was not significantly increased in patients with critical progression in our study cohort. Regarding age, there was an expected higher number of people in higher age groups that had to be hospitalized but comparing the progress and nonprogress group there was no significant difference at the age distribution between them. Comparing the number of hospitalized normal weight patients with that of overweight and obese patients, the latter was disproportionately higher as was expected. But within the group of hospitalized COVID-19 patients, they did not have a clear increased risk for a critical progression.

While on the one hand these undetected differences may be a weakness in the validity of our cohort, this observation may also strengthen our results, as they relate to a relatively even distribution of risk factors between the compared groups and reduce the influence of known risk factors in our analysis.

Also interesting is the observation that there was only 1 active smoker among our hospitalized COVID-19 patients. Similar observations were found in other studies, e.g., that the proportion of smokers among hospitalized patients was lower than the prevalence in the respective population would suggest [5]. There have been previous discussions nourished through other study results whether a protective value of nicotine, and thus smoking, could be presumed [6–8]. Ultimately, however, only insufficient robust data were available to derive any real recommendations from this information [9]. There are several peer-reviewed systematic reviews and meta-analyses that specifically address the question of whether active or past smoking is more protective or harmful. The underlying original work in these reviews overlaps to some extent and is predominantly of rather poor quality with heterogeneous results.

A peer-reviewed multicenter retrospective observational study [10] from Italy included 3894 patients who were hospitalized with confirmed SARS-CoV‑2 infection. The multivariable hazard ratio (HR) for active smoking as a risk of fatal outcome was 0.94 but was not significant (95% confidence interval [CI] 0.63–1.09). The effect of chronic lung diseases was also examined, and the HR indicated a small association with worse prognosis but was also not significant (HR 1.20; 95% CI 0.97–1.48). Patanavanich et al. [11] selected 19 peer-reviewed articles for their review, which included a total of 11,590 COVID-19 patients, predominantly hospitalized (outpatients considered in two studies). There was a low to moderate association (OR = 1.91; 95% CI 1.42–2.59) for a more severe COVID-19 course when a patient had a positive smoking history.

Eighteen peer-reviewed studies were analyzed in a review by Farsalinos et al. [5]. An analysis of the risk of experiencing a more severe disease course as an active smoker compared to the risk as a former smoker showed a just significant low to moderate association (OR 1.53; 95% CI 1.06–2.20).

A review by Reddy et al. [12] included data from 47 studies with a total of 32,849 hospitalized COVID-19 patients. Five of these studies had a prospective study design, and another 22 were rated as good quality. In the meta-analysis, active smokers showed a slightly to moderately increased risk of developing severe COVID-19 (risk reduction [RR] 1.8; 95% CI 1.14–2.85) or severe to critical COVID-19 (RR 1.98; 95% CI 1.16–3.38) than former and never smokers. An observed increased RR of mortality was not significant (RR 1.45; 95% CI 0.83–2.60). Comparing active and former smokers as one group on the one hand and never-smokers on the other, the results of the original studies were more consistent and showed a slightly increased risk in the smoking-history group for a severe, a severe or critical, or a fatal outcome (respectively OR [and 95% CI]: 1.31 [1.12–1.54]; 1.36 [1.19–1.53]; 1.26 [1.20–1.32]).

Limitations of this pilot study result on the one hand from the retrospective design, but also from the small number of cases as already mentioned before. Further studies with higher numbers of cases are necessary and could also further describe a correlation of the necessary oxygen flow per minute and a worse prognosis.

In conclusion, the qSOFA score should not be the only screening tool for a risk assessment of critical disease progression in COVID-19 patients. Indeed, patients with a score of ≥ 2 have a high risk of deteriorating and should receive intensive care or should at least be monitored closely. But critical progression also often occurred while the score was not able to give a corresponding hint, even shortly before those events. A more useful measurement seems to be the peripheral oxygen saturation to determine the need of oxygen supplementation (e.g., less than 94% SO_2_). Patients with a high qSOFA score or/and an impaired SO_2_ should be monitored more closely to initiate necessary intensive medical measures at an early stage, to look closer for affected organ systems other than the respiratory system (especially for affected renal function, neurologic impairments, and myocardial involvement) and thus to initiate the necessary instrumental diagnostics.
